# *Dvl3* polymorphism interacts with life events and pro-inflammatory cytokines to influence major depressive disorder susceptibility

**DOI:** 10.1038/s41598-018-31530-2

**Published:** 2018-09-21

**Authors:** Jian Zhang, Jiarun Yang, Dong Han, Xueyan Zhao, Jingsong Ma, Bo Ban, Xiongzhao Zhu, Yanjie Yang, Depin Cao, Xiaohui Qiu

**Affiliations:** 1 Psychology Department of the Public Health Institute of Harbin Medical University, Heilongjiang Province, Harbin, China; 2 Affiliated Hosptial of Jining Medical University, Shandong Province, Jining, China; 3 Medical Psychological Institute of the Second Xiangya Hospital of Central South University, Hunan Province, Changsha, China; 4 Harbin Medical University, Heilongjiang Province, Harbin, China

## Abstract

The purpose of this study is to explore *Dvl3* variants and their interaction with negative life events on MDD susceptibility in a Chinese Han population. Additionally, we also attempted to identify whether there is an association between *Dvl3* variants and pro-inflammatory cytokines. A total of 1102 participants, consisting of 550 patients with MDD and 552 healthy subjects, were recruited for genotyping by TaqMan allelic discrimination assay. Pro-inflammatory cytokine mRNA levels in peripheral blood were measured by QPCR. After the assessment of negative life events by the Life Events Scale, the *Dvl3* gene–environment interaction (G × E) and risk factors were evaluated using generalized multifactor dimensionality reduction method (GMDR) and logistic regression analysis, respectively. This study is the first to reveal the interaction between *Dvl3* allelic variations and negative life events as well as pro-inflammatory cytokines on MDD susceptibility in a Chinese Han population.

## Introduction

Major depressive disorder (MDD), as a severe psychiatric disorder featured by persistent low mood, has a serious effect on physical and psychological health in humans. The World Health Organization has predicted that MDD will be the chief disability-causing illness by 2030, with a high disease burden worldwide^1^. For evaluation and treatment of this condition, it is important to determine etiological factors and review advances in diagnostic modalities sensitive and specific to MDD. Emerging evidence has indicated that MDD has modest heritability (31–42%)^2^; therefore, a large number of genome-wide association studies (GWAS) have been conducted to identify candidate genes for this condition. Recently, two relatively large and comprehensive genomic and transcriptomic studies have implicated *Dvl3* as a significant risk gene for MDD^3,4^.

*Dvl3* is a gene located on chromosome 3q27. It codes for the disheveled segment polarity protein (Dvl)-3, which takes part in the wingless-related integration site (Wnt) signaling pathway, which is crucial for regulation of hippocampal neurogenesis^5^. The role of the Wnt signaling pathway in mood disorders was recently reviewed^6,7^, and Dvl was confirmed to be associated with neural development processes such as dendritic arborization^7^. Wilkinson *et al*. found that down-regulation or blockade of Dvl promoted depression-like behavior in depression models^8^. However, to date, there has been little research on the association between *Dvl* polymorphism and MDD. There has only been one mega-analysis of GWAS, which revealed a suggestive association between *Dvl3* polymorphism (rs1969253) and MDD in individuals of European ancestry (9240 patients with MDD and 9519 control subjects); however, the association did not reach genome-wide significance (P = 4.8 × 10^−6^)^4^. Subsequently, Jansen *et al*. provided further evidence that *Dvl3* has an effect on MDD. However, both these studies—which strongly indicated that *Dvl3* has an important role in MDD—were conducted among European populations. Therefore, whether there is an association between *Dvl3* and MDD among populations of other ethnicities has yet to be confirmed.

Since MDD is a mulitifactorial complex disease, accumulated evidence suggests that both environmental and genetic factors are involved in the etiology of MDD^9^. Caspi *et al*. initially provided the evidence regarding the key role of environmental factors in causing depression^10^; this was followed by several correlated clinical studies which supported the findings^11–13^. Negative life events, as one of the most well-established environmental risk factors for MDD, refer to loss, divorce, serious illness, interpersonal or family problems, relationships, and social difficulties^14^. The genetic variation modulates the individual sensitivity to negative environmental influences, making individuals not vulnerable to such events and others vulnerable. Studies on etiology of MDD have illustrated the gene–environment (G × E) interaction of certain risk genes and negative life events was associated with considerably greater risk of developing mood disorders. However, as far as we know, the interaction between *Dvl3* polymorphisms and negative life events on MDD susceptibility has not been investigated thus far.

Additionally, over the past several decades, there has been strong and wide support for the cytokine hypothesis, a general hypothesis based on immune–inflammatory system dysfunction. It is one of the more prevalent theories concerning MDD and might provide insights into the pathogenesis of depression and development of biomarkers and, ultimately, more effective depression therapies. The hypothesis^15^ regards MDD as an environment–neuro–immune disorder. It is believed that physiological or psychological stress could activate the immune system, cause abnormal cytokine production, and thus affect the central nervous system, resulting in emotional changes or disease. Indeed, there is substantial evidence linking MDD to alterations in the inflammatory system, including the presence of elevated levels of Pro-inflammatory cytokines together with other mediators of inflammation^16–18^. However, to date, little is known about the effect of associations between *Dvl* variants and immune system responses on MDD.

Therefore, we conducted a case-control study to ascertain whether *Dvl3* polymorphisms and negative life events as well as their interactions were associated with MDD among northern Chinese Han population. Moreover, considering that the etiology of depression is associated with stress, we also analyzed inflammatory cytokine production in peripheral blood in patients with MDD and discussed the potential connection between inflammatory cytokines and MDD.

## Results

### Demographic characteristics of the participants

Basic characteristics of participants were displayed in Table 1. No significant difference was found in age, gender or marital status. The mean ages of the patients and control subjects were 44.53 ± 13.53 and 43.19 ± 9.08 years, respectively.

**Table 1:** Demographic characteristics of participants.

Variables	MDD (n = 550)	Control (n = 552)	χ^2^/t	P value
Age, years	44.53 ± 13.53	43.19 ± 9.08	−1.938	0.053
Sex			3.553	0.059
Male	165 (30.00%)	195 (35.30%)		
Female	385 (70.00%)	357 (64.70%)		
Marital status			5.272	0.261
Single	79 (14.40%)	65 (11.80%)		
Married	436 (79.30%)	459 (83.20%)		
Divorced	31 (5.60%)	21 (3.80%)		
Widowed	4 (0.70%)	7 (1.30%)		
Negative LES score	9.66 ± 18.37	2.68 ± 6.78	−8.358	3.45E-16

Data are presented as mean ± standard deviation or number (percentage).

### Single-marker association with MDD

The genotypic distributions of all selected *Dvl3* polymorphisms conformed to the Hardy–Weinberg equilibrium (P > 0.05). The genotypic distribution pattern and allelic frequencies of the two SNPs among MDD and controls were shown in Table 2. After Bonferroni correction, significant differences in genotypic between the patients and controls were confirmed in rs1709642 (χ^2^ = 19.464; P = 5.95E-05), and the association between rs1969253 and MDD was not significant. While there was a significant allelic association between rs1709642 (χ^2^ = 11.320; P = 0.001; OR = 1.333; 95% CI = 1.127–1.576) and MDD, the corresponding association between rs1969253 and MDD was not significant (P = 0.055).

**Table 2:** Genotype distribution patterns and allelic frequencies of *Dvl* polymorphisms among patients with MDD and control subjects.

SNP	Sample	Genotype (%)			P	Allele (%)		P	Odds ratio (95% CI)
rs1969253		CC	CA	AA		C	A		
	MDD	123 (22.4)	302 (54.9)	125 (22.7)	0.056	548 (49.8)	552 (50.2)	0.055	1.177
	Control	162 (29.3)	271 (49.1)	119 (21.6)		595 (53.9)	509 (46.1)		(0.996–1.392)
rs1709642		CC	CT	TT		C	T		
	MDD	107 (19.5)	313 (56.9)	130 (23.6)	**1.19E-04**	527 (47.9)	573 (52.1)	**0.002**	1.333
	Control	171 (31.0)	266 (48.2)	115 (20.8)		608 (55.1)	496 (44.9)		(1.127–1.576)

Bold significant *p*-values were corrected by Bonferroni correction.

### Gene–environment interaction analysis on MDD

The gene–environment interaction effects in MDD susceptibility were detected using a GMDR model with age, smoking and alcohol consumption as covariates. Results of the GMDR analysis are summarized in Table 3. The prediction accuracy *p*-value was determined using the permutation test with 1000 replications. Significant two-locus and three-locus interaction models were observed (*p* < 0.05). The interaction between the two SNPs relative to negative life events showed a CV consistency of 10/10 and a testing accuracy of 65.74%. As results suggested that there was a significant interaction between *Dvl3* polymorphisms and negative life events on MDD susceptibility among the Chinese Han population, this was considered to be the best multi-locus model.

**Table 3:** The best gene–environment interaction models obtained by GMDR.

Locus No.	Best models	PE (%)	CV	P value
2	rs1969253, negative life events	35.26	10/10	<0.001
3	rs1969253, rs1709642, negative life events	34.26	10/10	<0.001

PE, prediction error; CV, cross validation.

Furthermore, we assessed the causative factors selected by GMDR using logistic regression analysis which incorporated age, smoking and alcohol consumption as covariates. Results were summarized in Table 4. Individuals with allele A^−^(CC) (OR = 1.927; 95% CI = 1.072–3.464) or A^+^(AC, AA) (OR = 2.518; 95% CI = 1.266–5.008) of rs1969253 and demonstrating a high negative life events score were in higher risk of developing MDD compared to the others. While subjects carrying the T^+^ allele (CT, TT) of rs1709642 with a high negative life events score (OR = 3.025; 95% CI = 1.471–6.223) were more susceptible to MDD relative to the rest of the study population.

**Table 4:** Interaction between *Dvl3* polymorphisms and negative life events.

Variables	MDD	Control	OR(95% CI)	P value
**A** ^**−**^ **rs1969253**				
and LN	91	137	1	
A^−^ and HN	32	25	**1.927 (1.072–3.464)**	**0.028**
A^+^ and LN	254	342	1.118 (0.819–1.526)	0.481
A^+^ and HN	173	48	**2.518 (1.266–5.008)**	**0.008**
**rs1709642**				
T^−^ and LN	84	146	1	
T^−^ and HN	23	25	1.599 (0.854–2.992)	0.142
T^+^ and LN	261	333	1.362 (0.996–1.864)	0.053
T^+^ and HN	182	48	**3.025 (1.471–6.223)**	**0.003**

LN, low negative life events; HN, high negative life events.

### Pro-inflammatory cytokines in peripheral blood

**Figure 1:**
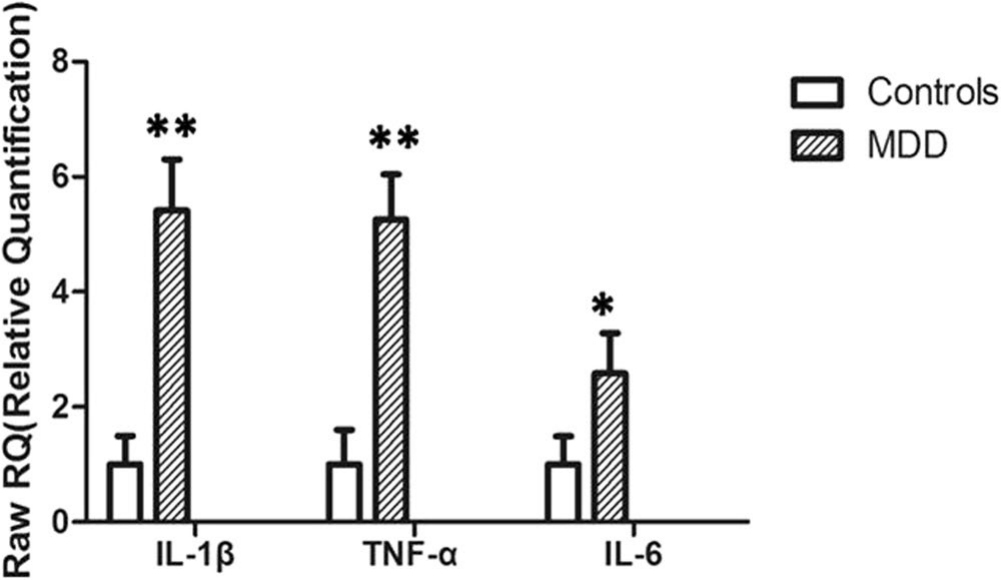
mRNA levels of pro-inflammatory cytokines (mean ± standard deviation) in peripheral blood in patients with MDD and control subjects (*n* = 100, each). **P < 0.01; *P < 0.05.

**Figure 2:**
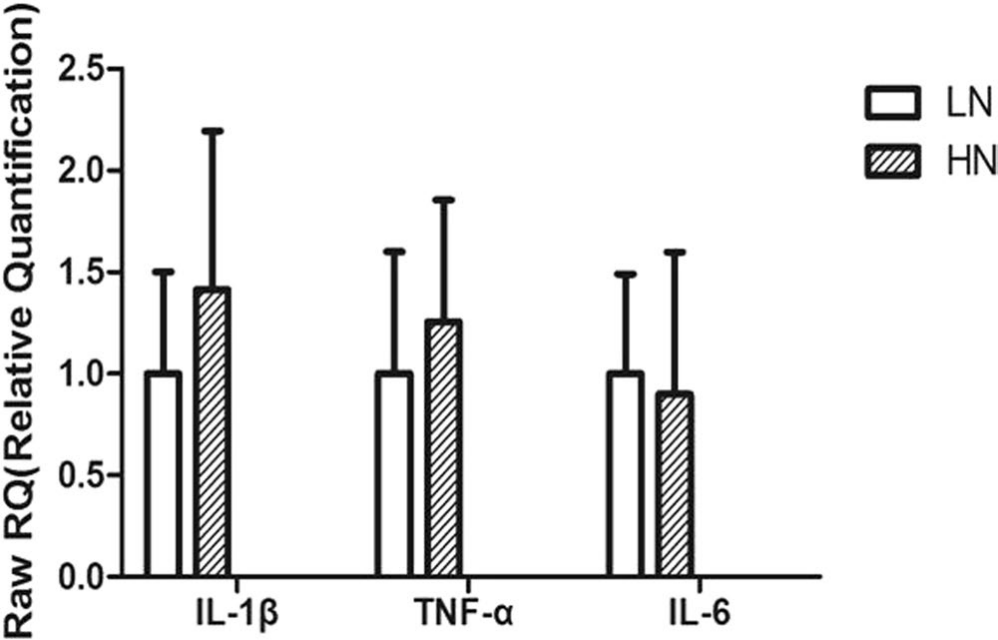
mRNA levels of pro-inflammatory cytokines (mean ± standard deviation) among patients with MDD with LN and HN (*n* = 100, each). LN, low negative life events; HN, high negative life events (*n* = 100).

**Figure 3:**
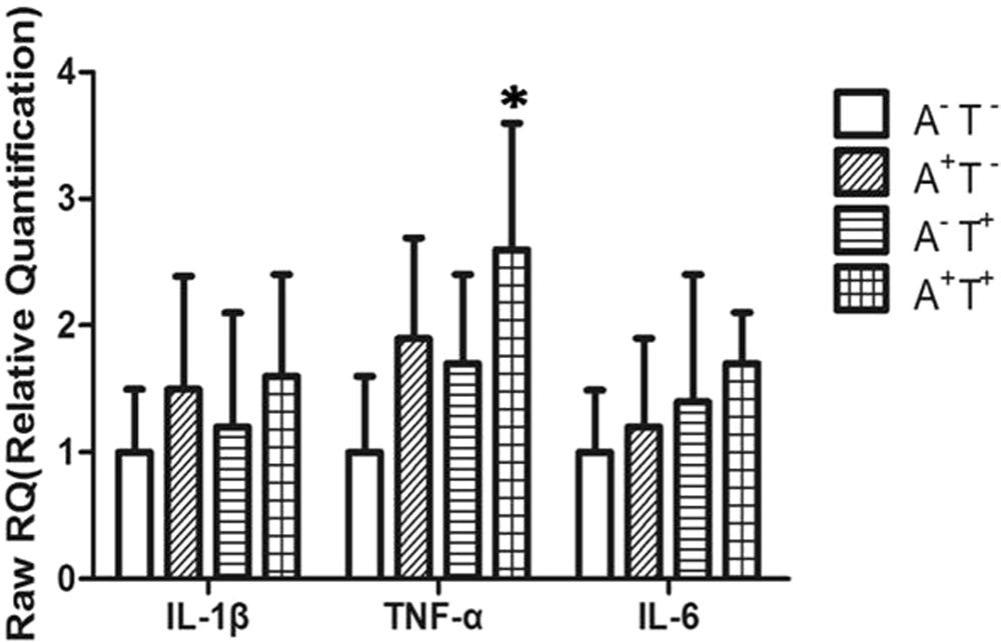
mRNA levels of pro-inflammatory cytokines (mean ± standard deviation) among patients with major depressive disorder with different *Dvl3* polymorphisms. A^−^, allele CC of rs1969253 (*n* = 25); A^+^, allele AC, AA of rs1969253 (*n* = 25); T^-^, allele CC of rs1709642 (*n* = 25); and T^+^, allele CT, TT of rs1709642 (*n* = 25). *P < 0.05.

First, upon intergroup comparison of mRNA expression levels in peripheral blood, patients with MDD were found to exhibit significantly higher mRNA levels of Pro-inflammatory cytokines IL-1β, TNF-α, and IL-6than the control subjects (Fig. 1). Second, in the MDD group, no difference was observed in mRNA expression levels of IL-1β, TNF-α, orIL-6 between patients with low and high negative life events (Fig. 2). Finally, upon comparing Pro-inflammatory cytokine mRNA levels among patients with MDD with different *Dvl3* polymorphisms, individuals carrying alleles A^+^(AC, AA) of rs1969253 and T^+^(CT, TT) of rs1709642 were found to exhibit significantly higher TNF-α mRNA levels than the remaining patients (Fig. 3).

## Discussion

In 2013 a PGC GWAS for MDD firstly threw a light on *Dvl3* and caught our attention, but the following studies by PGC GWAS did not verify the significance of *Dvl3*. Whereas Jansen *et al*. provided further evidence^19^ that *Dvl3* has an effect on MDD. In the present study, we attempted to investigate the role of *Dvl3* in susceptibility to MDD among the Chinese Han population. We found significant differences in genotypic and allelic frequencies of *Dvl3* polymorphisms between patients with MDD and control subjects and also confirmed one possible susceptibility loci—rs1709642—for onset of MDD. After Bonferroni correction, the current study found no association of *Dvl3* rs1969253 with MDD at the single-locus level. According to our results, individuals with genotypes T^+^ (CT, TT) of rs1709642 when exposed to high negative life events were more likely to suffer from MDD than the remaining subjects. Similarly, in a recent gene expression study (MDD, 882; remitted MDD, 635; and control, 331), Jansen *et al*. observed that *Dvl3* expression was up-regulated among patients with current MDD. Another larger gene expression study reported that *Dvl3* polymorphism (rs1969253) was associated with MDD among individuals of European ancestry, although the association failed to reach genome-wide significance. We suspected that the inconsistent result may be arisen due to sample differences: the number of MDD attacks, the proportion of melancholic MDD and different stages of MDD, for example. In addition, it is still unclear to which differences in sample immune response^20,21^, neuroplasticity, or other factors contribute to the failure to replicate genetic effects of *Dvl3* gene in the CONVERGE sample. Some scholars^22^ proposed lack of consideration of such environmental effects may be one prominent reason for the failure of replication in candidate gene studies and GWAS into the genetic background of depression.

Studies targeted depression as a product of multiple networks, where the activity of the pathways may be influenced by both genetic disposition and environmental exposure, mostly by individual-specific environmental factors^23,24^. Response to stress is regarded as one of the most important hypotheses for the etiology of MDD^25^. Existing literature confirms that negative life events are closely related to the incidence and severity of MDD^26^. However, thus far, no information on G × E interaction of *Dvl3* in the risk of developing MDD has been found. Our results revealed significant interaction effects between *Dvl3* polymorphisms and negative life events with regard to the risk of developing MDD. We observed two best models: a two-factor model indicating interactions between rs1969253 and negative life events and a three-factor model indicating interactions betweenrs1969253–rs1709642 and negative life events. This result helps explain why our study just like the previous study^4^ on *Dvl3* polymorphism rs1969253 and MDD failed to achieve genome-wide significance —environmental factors such as negative life events should be considered jointly with genetic polymorphisms. The present result regarding rs1709642 is an important discovery which remains to be further verified. For all we know, this study is the first to survey the impact of interactions between allelic variations in *Dvl3* and negative life events on the susceptibility of MDD.

Consistent with previous findings, *Dvl3* has emerged as a promising genetic risk factor for MDD in the present study. It has been reported that *Dvl3* transcript levels are down-regulated in the nucleus accumbens and frontal region^8,27^ in individuals with MDD and up-regulated in leukocytes in individuals reporting social isolation^28^. There is evidence that *Dvl* influences MDD both through intermediate *Dvl3* expression in the brain and the Wnt or NF-κB (nuclear factor kappa-light -chain-enhancer of activated B cells) pathway^29,30^, which is closely related with neuro-immune interactions. There are now multiple lines of evidence indicating that pro- and/or anti-inflammatory cytokine levels are altered in the serum or cerebrospinal fluid^31,32^ in patients with depression. The statement that IL-1β, IL-6, and TNF-α levels are increased in the serum and/or plasma in patients with depression^33–36^ is widely accepted to date. For this reason, in the present study, we attempted to identify whether there is an association between *Dvl3* variants and Pro-inflammatory cytokines. Another remarkable finding of the present study is the association between *Dvl3* variants and inflammatory response. Genetic polymorphism of *Dvl3* (rs1969253 and rs1709642) was associated with increased TNF-α levels. This effect was independent of a history of negative life events. Although the patho-physiological role of TNF-α in MDD is not clear, we could exclude the possibility that *Dvl3* mutations influence TNF-α level through the NF-κB pathway. There has been substantial research on the interconnectedness of NF-κB and TNF, including some studies involving knockout mouse models^37^. Although *Dvl3* is not likely to be a major susceptibility factor for MDD, its clinical value might not be negligible. Analysis of *Dvl3* polymorphisms might help identify groups of patients with MDD who are likely to respond to anti-inflammatory agents.

The present results should be considered on the basis of several limitations. First, our selected SNPs were located in the same block of *Dvl* and might not be representative of genetic information in other regions of the gene. Second, the results of life events assessment using LES, to some extent, might be influenced by subjective interpretation. Third, this study merely included patients and control subjects of Chinese Han origin from Northern China, and the sample size was relatively small. It is, therefore, difficult to generalize these results to other populations.

## Materials and Methods

### Participants

A total of 1102 participants from a single hospital, which consisted of 550 first-episode depressive patients with MDD and 552 healthy control subjects, were recruited for the study between February 2014 and December 2016. All participants were of Chinese Han origin and were living in the same geographical area in the north of China. Basic characteristics of participants were displayed in Table 1. Cases were diagnosed with MDD by at least two psychiatrists according to the Fourth Edition of the Diagnostic and Statistical Manual of Mental Disorders (DSM-IV)^34,36–38^. The patients all had first-episode depressive disorder and not a single patient received any antidepressant treatment within 4 weeks preceding assessment. Only patients with a minimum HAMD score of 21 were included in this study.

Subjects with a history of brain organic mental disorders, a family history of genetic disease, mental retardation or dementia, immunodeficiency disease, or those who recently received blood transfusion treatment were excluded from the study. Healthy subjects with a family history of mental disorders were also excluded from participating. Subjects who failed to provide sufficient information or continue the study prematurely were also excluded. All procedures of our research were conducted according to the guidelines established by the National Institutes of Health, and every effort was made to minimize suffering. This study was approved by Harbin Medical University Research Ethics Committee, Harbin, China. Written informed consent forms were obtained from all participants in our study.

### Outcome measures

#### Evaluation of negative life events

The Life Events Scale (LES) developed by Yang and Zhang was used to evaluate negative life events, which includes 48 items classified into 3 domains: family life (28 items), work (13 items), and social and other aspects (7 items). The validity of LES has previously been confirmed in a Chinese population^39^. The negative events mainly refer to serious illness, relationship breakdown, housing difficulties, unemployment, social difficulties, and financial crises. The LES was filled by interviewers according to their own experience and the scores for positive, negative and total life events were calculated. The scale assesses four aspects of the event: time of occurrence (absent = 1; over a year ago = 2; within the past year = 3; or chronic = 4), character (good = 1 or bad = 2), influence on mood (absent = 1; mild = 2;moderate = 3; severe = 4; or extreme = 5), and duration of influence (≤3 months = 1; 3–6 months = 2; 6–12 months = 3; or >12 months = 4). The 75% percentile (a score of 6) was considered as the cutoff value for high and low level categories.

#### DNA extraction and genotyping methods

Genomic DNA was isolated from collected EDTA-anticoagulated blood samples using the AxyPrepTM Blood Genomic DNA Miniprep Kit (Axygen, Union City, CA, USA). Using the Haploview program^40^, we selected two tag single-nucleotide polymorphisms (SNPs; rs1969253 and rs1709642) from the whole *Dvl* gene. Primers for polymerase chain reaction (PCR) amplification were designed with Primer 5.0 software (Premier, Canada), and final specific primers were checked using NCBI-BLAST (http://www.ncbi.nlm.nih.gov/). The ABI PRISM 7900 Sequence Detection System (Applied Biosystems, Foster City, CA, USA) and ABI 3730 DNA sequencer (Applied Biosystems) were used for genotyping and purifying.

#### Quantitative PCR (qPCR)

Messenger RNA (mRNA) expression levels of interleukin (IL)-1β, tumor necrosis factor (TNF)-α, andIL-6 were determined by qPCR assay using SYBR Green PCR Master mix reagent (TaKaRa, Otsu, Shiga, Japan). Briefly, RNA was extracted using a blood/liquid sample total RNA rapid extraction kit (BioTeke Corporation, Beijing, China). First-strand cDNA synthesis was performed with 1 μg of total RNA usingthe PrimeScript^TM^ RT reagent kit (Takara, Dalian, China) following the manufacturer’s instructions. The quality and quantity of total RNA and genomic DNA were evaluated by 1.5% agarose gel electrophoresis and measured with a UVS-99 densitometer (ACTGene, Piscataway, NJ, USA). Relative expression levels were normalized against β-actin and analyzed by the 2^-ΔΔ^Ct method [ΔΔCt = (Ct Target - Ct Reference) sample - (Ct Target- Ct Reference) control].

### Statistical analysis

Pearson’s chi-square test was used to perform Hardy–Weinberg equilibrium tests, and pairwise linkage disequilibrium. Using SPSS version 20.0 (International Business Machines, Armonk, NY, USA), the Student’s t-test was used to compare the means of continuous variables between cases and controls, and the differences in the distribution of categorical variables were evaluated using the chi-square test. Bonferroni correction was applied to the *p*-value to correct for multiple testing and a corrected *p*-value values ≤ 0.05 (two-tailed) was considered as being statistically significant.

Gene–environment interactions were analyzed using generalized multifactor dimensionality reduction (GMDR) software (version 1.0.0) which was designed by the Computational Genetics Laboratory, Dartmouth Medical School, Lebanon, NH, USA^41^. The best gene-environment interaction model based on the values arising from cross-validation (CV) consistency and accuracy testing were selected. A permutation test with 1000 replications was used to measure empirical *p*-values thereby substantiating the significance of the model. In GMDR analysis, a *p*-value was corrected for multiple testing by permutation test and a corrected *p*-value < 0.05 (two-tailed) was considered to be statistically significant

For validating the results of GMDR, odds ratios (OR; with 95% confidence intervals) of risk factors were computed by logistic regression analysis using SPSS version 20.0. To narrow down the number of possible combinations, only dominant models were subjected to further analysis. The Bonferroni method was used to correct P values for multiple comparisons.

## Conclusion

For all we know, this is the first report that there was an effect modification between *Dvl3* variation and negative life events on MDD susceptibility in a Chinese Han population. This study is also the first to reveal the underlying genetic architecture of *Dvl3*, including genetic loci frequencies, effect sizes and models of action. The candidate SNPs could regulate *Dvl3* expression, and even modulate the effects of negative life events; therefore, their effects converged to a signifcant modulation on the development of MDD. These factors are important determinants for the success in identifying genetic associations for disease(s) with complex traits. Although their specific mechanism still needs further study, the present results confirm *Dvl3* as a susceptibility factor for MDD as well as a modifier of inflammatory response.
